# Nomogram incorporating TyG index and TG/HDL ratio for early prediction of gestational diabetes mellitus

**DOI:** 10.1186/s12884-026-08732-y

**Published:** 2026-02-18

**Authors:** Haoyi Jia, Siyu Liu, Mengjie zhou, Yannan Cao, Xupei Gan, Pengyuan He, Feifei Li, Yanhua Xu, BingXin Wang, Xianming Xu

**Affiliations:** 1 Department of Obstetrics and Gynecology, Shanghai General Hospital, Shanghai Jiao Tong University School of Medicine, No. 165 Xinsongjiang road, Songjiang District, Shanghai, 201600 People’s Republic of China; 2 Department of Urology, Shanghai General Hospital, Shanghai Jiao Tong University School of Medicine, Shanghai, 201600 People’s Republic of China; 3 Department of Obstetrics and Gynecology, Songjiang Maternal and Child Health-Care Hospital, Shanghai, 201600 People’s Republic of China

**Keywords:** Gestational diabetes mellitus, TyG index, TG/HDL ratio, Insulin resistance, Prediction model

## Abstract

**Background:**

The triglyceride-glucose (TyG) index and the triglyceride-to-HDL cholesterol (TG/HDL) ratio have emerged as surrogate markers of insulin resistance, but their predictive value for gestational diabetes mellitus (GDM) remains uncertain.

**Methods:**

This retrospective cohort study included 4,239 pregnant women, among whom 919 developed GDM and 3,320 had normal glucose tolerance. Demographic, anthropometric, and biochemical parameters were collected during early pregnancy, specifically at 8–13 gestational weeks (first trimester), OGTT-related glucose and insulin measurements (FIN, 1hPIN, 2hPIN) were obtained later at 24–28 weeks during the routine 75-g OGTT, after at least 8 hours of overnight fasting. Logistic regression, restricted cubic spline models, correlation analysis, age-stratified analyses, and mediation analysis were performed to evaluate associations of TyG and TG/HDL ratio with GDM. A nomogram model was constructed and internally validated using a random training/validation split.

**Results:**

Higher TyG and TG/HDL ratio levels were independently associated with increased GDM risk (TyG: aOR 4.07, 95% CI 3.34–4.95; TG/HDL ratio: aOR 1.78, 95% CI 1.56–2.03). Women with both elevated TyG and TG/HDL ratio showed the highest risk (aOR 2.11, 95% CI 1.77–2.52). The nomogram demonstrated modest discrimination, with an AUC of 0.657 in the training set and 0.648 in the internal validation set, the latter representing the primary performance estimate. Calibration was satisfactory in both sets.

**Conclusions:**

Early-pregnancy lipid–glucose dysregulation, reflected by higher TyG and TG/HDL ratio levels, is associated with subsequent GDM development. Although the predictive performance is modest, the combined indices may support exploratory early-pregnancy risk stratification.

**Supplementary Information:**

The online version contains supplementary material available at 10.1186/s12884-026-08732-y.

## Background

Gestational diabetes mellitus (GDM) is a common pregnancy complication characterized by glucose intolerance first recognized during gestation, affecting approximately 5–18% of pregnancies globally depending on population and diagnostic criteria [1, 2]. It is associated with increased risk of adverse maternal and neonatal outcomes, including preeclampsia, macrosomia, cesarean delivery, and long-term metabolic disorders such as type 2 diabetes and cardiovascular disease in both mothers and offspring [3, 4]. Moreover, GDM-related macrosomia has been associated with unique ultrastructural alterations in the placenta, underscoring the impact of maternal metabolic disturbances on placental morphology and fetal growth [5].

The pathophysiology of GDM is multifactorial, with insulin resistance and β-cell dysfunction as central features [6]. Obesity and advanced maternal age have been consistently identified as major risk factors; however, the role of early metabolic disturbances, particularly dyslipidemia and insulin resistance in the first trimester, remains underexplored [7]. The identification of reliable early biomarkers is essential for timely risk stratification and preventive intervention.

Recent attention has focused on lipid-related surrogate markers that integrate glycemic and lipid metabolic pathways. Among these, the triglyceride-glucose (TyG) index, calculated from fasting triglycerides and glucose, has emerged as a proxy for insulin resistance and a potential early indicator of metabolic dysfunction [8, 9]. Similarly, the triglyceride-to-high-density lipoprotein cholesterol ratio (TG/HDL ratio) has been associated with adverse cardiovascular and glycemic outcomes, reflecting atherogenic dyslipidemia and impaired lipid clearance [10, 11].

Several studies have shown that TyG and TG/HDL ratio are associated with insulin resistance and metabolic syndrome in the general population [12, 13]. However, their utility in predicting GDM, especially in early pregnancy, has not been fully validated across diverse cohorts. A few recent investigations suggest that these indices may offer superior or complementary predictive value compared to traditional parameters like fasting glucose or BMI alone [14].

Rather than proposing a fundamentally new concept, this study aims to confirm previously reported associations between TyG, TG/HDL ratio and GDM risk in a large Chinese cohort, and to explore the additional information provided by their combined categorization and age-stratified patterns. These analyses are intended to provide population-specific evidence and exploratory insights, rather than to claim a major conceptual innovation. Understanding the early metabolic signatures of GDM could enhance predictive modeling and inform targeted interventions during critical windows of fetal development.

## Methods

### Study population and design

A total of 4,239 pregnant women were enrolled in this study, which included individuals attending routine prenatal care at Shanghai General Hospital between March 2021 and March 2023 (Figure S1). Baseline demographic and fasting biochemical parameters (FPG, TG, TC, HDL, LDL, TyG, TG/HDL ratio, TyG-BMI) were collected at 8–13 gestational weeks during the first antenatal visit after an overnight fast of at least 8 h. In contrast, OGTT-related glucose and insulin measurements (FIN, 1hPIN and 2hPIN) were obtained at 24–28 gestational weeks during the standard 75 g OGTT for GDM diagnosis. All blood samples were obtained in the morning after at least 8 h of fasting, and biochemical analyses were performed in a central certified laboratory. Participants were included based on the following criteria: (1) availability of complete baseline demographic data and key biochemical measurements during pregnancy; and (2) no history of long-term use of medications known to interfere with glucose metabolism, such as glucocorticoids, hormonal contraceptives, or adrenal inhibitors.

Exclusion criteria comprised: (1) conception via assisted reproductive technologies or multiple gestations; (2) a prior or current diagnosis of type 1 or type 2 diabetes mellitus; (3) presence of malignant tumors, infectious or inflammatory diseases (acute or chronic), hepatic dysfunction, or inherited lipid disorders; and (4) cases involving medical termination of pregnancy or intrauterine fetal demise.

For subgroup analysis, participants were stratified according to a dual-index approach using the median values of the triglyceride-glucose (TyG) index (cut-off: 8.5) and triglyceride-to-HDL cholesterol (TG/HDL) ratio (cut-off: 0.8). Based on these thresholds, the cohort was divided into four metabolic phenotype groups: (Group 1: TyG < 8.5 and TG/HDL ratio < 0.8; Group 2: TyG ≥ 8.5 and TG/HDL ratio < 0.8; Group 3: TyG < 8.5 and TG/HDL ratio ≥ 0.8; Group 4: TyG ≥ 8.5 and TG/HDL ratio ≥ 0.8).

Baseline demographic and metabolic parameters were compared across these groups to evaluate associations with subsequent gestational diabetes mellitus (GDM) development.

### Definition of GDM

Gestational diabetes mellitus (GDM) is commonly diagnosed between the 24th and 28th weeks of pregnancy using the oral glucose tolerance test (OGTT). After an overnight fast, the patient consumes 75 g of glucose, and plasma glucose levels are assessed at three time points: prior to ingestion (fasting), and at 1 h and 2 h post-glucose load. According to the diagnostic criteria established by the International Association of Diabetes and Pregnancy Study Groups (IADPSG), GDM is diagnosed if any of the following thresholds are met: Fasting glucose (0 h) ≥ 92 mg/dL (5.1 mmol/L), 1-hour glucose (1 h) ≥ 180 mg/dL (10.0 mmol/L) and 2-hour glucose (2 h) ≥ 153 mg/dL (8.5 mmol/L) [15, 16]. These cut-off values are widely accepted and endorsed by several international guidelines for the identification of GDM during mid-pregnancy.

### Definitions of TyG, TG/HDL ratio and related indices

All fasting biochemical measurements used to calculate TyG, TyG-BMI and TG/HDL ratio (FPG, TG, TC, HDL and LDL) were obtained from early-pregnancy (8–13 weeks) morning blood samples after an overnight fast of at least 8 h. By contrast, fasting and post-load insulin measurements (FIN, 1hPIN and 2hPIN) and OGTT glucose values were obtained during the 24–28-week OGTT. Triglycerides (TG), total cholesterol (TC), HDL, LDL and fasting plasma glucose (FPG) were measured in SI units (mmol/L). The triglyceride-glucose (TyG) index is widely used as a reliable surrogate marker of insulin resistance (IR), derived from fasting plasma glucose (FPG) and triglyceride (TG) concentrations [17]. The formula is: TyG = ln [TG (mg/dL) × FPG (mg/dL) / 2] [18]. Because the laboratory reported TG and FPG in mmol/L, these values were internally converted to mg/dL using standard conversion factors (TG: mmol/L × 88.57; glucose: mmol/L × 18) exclusively for the computation of TyG and QUICKI. All glucose and lipid values presented in the tables and text remain in mmol/L. To enhance predictive accuracy, TyG-BMI was computed as TyG × BMI (kg/m²) [19]. The TG/HDL ratio was calculated using TG (mmol/L) ÷ HDL (mmol/L); HDL originally provided in g/L was converted to mmol/L using standard factors to ensure consistent units [20].

In addition, several insulin resistance and β-cell function indices derived from the Homeostasis Model Assessment (HOMA) are commonly applied: HOMA-IR = [FPG (mmol/L) × Fasting Insulin (µU/mL)] / 22.5 [21]. HOMA-IS = 20 × Fasting Insulin (µU/mL) / [FPG (mmol/L) − 3.5] [22]. HOMA-β = [Fasting Insulin (µU/mL) × FPG (mmol/L)] / [2hPG + 1hPG − 2 × FPG] [23]. The Quantitative Insulin Sensitivity Check Index (QUICKI), is expressed as: QUICKI = 1 / [log(Fasting Insulin (µU/mL)) + log(FPG (mg/dL))], where FPG in mmol/L was internally converted to mg/dL using the factor ×18. All lipid parameters (TG, TC, HDL, LDL) and FPG are consistently expressed in mmol/L throughout the manuscript and tables, except where internal unit conversion was required to calculate TyG and QUICKI [24]. 

### Assessment of covariates

Based on previous literature on gestational diabetes mellitus, we first considered the following variables as potential covariates: maternal age, pre-pregnancy body mass index (BMI), gravidity, parity, and selected biochemical markers measured in early pregnancy (fasting plasma glucose [FPG], triglycerides [TG], total cholesterol [TC], high-density lipoprotein cholesterol [HDL], low-density lipoprotein cholesterol [LDL] and the TyG-BMI index) and OGTT-related insulin indices measured at 24–28 weeks (FIN, 1hPIN, 2hPIN). For the main multivariable models, we adjusted for maternal age, pre-pregnancy BMI, gravidity, and parity, which are well-established GDM risk factors that were available for all participants and are not considered intermediate variables on the causal pathway between early-pregnancy lipid–glucose indices and GDM. Information on other important GDM determinants, such as family history of diabetes, history of GDM in previous pregnancies, detailed lifestyle factors (dietary patterns, physical activity, smoking, and alcohol use), and socioeconomic indicators (education, income, occupation), was not systematically recorded in the electronic medical record system or contained substantial missing data, and therefore could not be reliably included in the multivariable models. We acknowledge this as a source of potential residual confounding and address it in the Discussion. BMI was calculated as weight (kg) divided by the square of height (m²), in accordance with the World Health Organization definition [17, 21, 25].

### Statistical analysis

Statistical analyses were performed using SPSS 27 (IBM Corp., Armonk, NY, USA) and R 4.1.2 (R Foundation for Statistical Computing, Vienna, Austria). Continuous variables were summarized as median (IQR) and compared using the Mann–Whitney U test, while categorical variables were expressed as n (%) and compared using the chi-square test. Associations between clinical/biochemical markers and GDM were evaluated using logistic regression, with results presented as odds ratios (ORs) and 95% confidence intervals (CIs) [26].

Three prespecified multivariable models were constructed using maternal age, pre-pregnancy BMI, gravidity and parity as adjustment covariates. Model 0 included only these covariates; Model 1 additionally included the TyG index; and Model 2 further added the TG/HDL ratio. Early-pregnancy fasting glucose and individual lipid fractions were not included in the main adjusted models to avoid overadjustment, as they are strongly correlated with TyG and TG/HDL ratio and may lie on the causal pathway. Stratified analyses were conducted by maternal age. A mutual statistical-dependency (variance-partitioning) analysis was performed to describe the shared triglyceride-related contribution between TyG and TG/HDL ratio. Because both indices were measured cross-sectionally and are algebraically related, this analysis does not meet causal-mediation assumptions and is interpreted purely descriptively [27].

For prediction modelling, a multivariable logistic regression including maternal age, pre-pregnancy BMI, gravidity, parity, TyG and TG/HDL ratio was developed and presented as a nomogram. The full cohort comprised 4,239 women (919 GDM cases), providing ample events per variable. The dataset was randomly split into a 70% training cohort and a 30% internal validation cohort. Discrimination was assessed using the area under the receiver operating characteristic curve (AUC); calibration was evaluated using calibration plots and the calibration slope with 1,000-bootstrap optimism correction; and decision curve analysis was used to assess net clinical benefit. The AUC from the validation cohort (0.648) was taken as the primary measure of model discrimination. A two-sided *P* < 0.05 was considered statistically significant.

## Result

The baseline characteristics of study participants, including demographic and metabolic parameters, are summarized in Table 1. Among the 4,239 pregnant women enrolled, those who developed GDM (*n* = 919) were slightly older and had a higher pre-pregnancy BMI compared with the non-GDM group (*P* < 0.001 for both). Biochemical profiling revealed that the GDM group exhibited a more adverse metabolic status, characterized by elevated fasting plasma glucose, triglycerides and LDL (all *P* < 0.001).

**Table 1:** Basic characteristics of study participants

Variables		GDM group (*n* = 919) M(P25,P75)/*N*(%)	NGT group (*n* = 3320) M(P25,P75)/*N*(%)	*P*-value
Demographic characteristics				
Maternal age(years)		31.00(28.00,34.00)	30.00(27.00,33.00)	< 0.001***
Pre-BMI(kg/m2)		24.21(21.88,27.33)	23.53(20.81,26.67)	< 0.001***
Gravidity	1	300(32.6)	1211(36.5)	0.006**
	2	274(29.8)	1028(31.0)	
	≥ 3	345(37.6)	1081(32.5)	
Parity	0	316(34.4)	1044(31.4)	0.208
	1	395(43.0)	1523(45.9)	
	≥ 2	161(22.6)	753(22.7)	
Biochemical indicators				
FPG(mmol/L)		4.84(4.51,5.20)	4.50(4.22,4.77)	< 0.001***
TG(mmol/L)		1.50(1.19,1.92)	1.32(1.06,1.68)	< 0.001***
TC(mmol/L)		4.84(4.34,5.42)	4.70(4.22,5.26)	< 0.001***
HDL(mmol/L)		1.61(1.36,1.85)	1.61(1.39,1.86)	0.416
LDL(mmol/L)		2.41(2.00,2.88)	2.30(1.92,2.76)	< 0.001***
TyG		8.65(8.42,8.93)	8.47(8.23,8.72)	< 0.001***
TyG-BMI		209.91(189.09,240.14)	199.20(175.36,225.98)	< 0.001***
TG/HDL ratio		0.93(0.71,1.28)	0.82(0.62,1.09)	< 0.001***

pre-BMI, pre-pregnancy body mass index; Gravidity, number of pregnancies; Parity, number of previous births at ≥ 24 gestational weeks. All lipid parameters (TG, TC, HDL, LDL) and FPG are expressed in mmol/L. TG/HDL ratio is unitless

*P < 0.05, **P < 0.01, ***P < 0.001

Notably, lipid-related indices including TyG and TG/HDL ratio were markedly higher in women with GDM than in those without (both *P* < 0.001). These findings suggest that early-pregnancy lipid–glucose dysregulation, reflected by TyG and TG/HDL ratio, may serve as potential risk factors for subsequent development of GDM.

In multivariable logistic regression models adjusted for maternal age, pre-pregnancy BMI, gravidity, and parity, both the triglyceride-glucose (TyG) index and the TG/HDL ratio exhibited strong associations with GDM. Specifically, after adjustment for maternal age, pre-pregnancy BMI, gravidity, and parity, the TyG index remained significantly associated with GDM (aOR = 4.07, 95% CI 3.34–4.95). The TG/HDL ratio also showed a significant association (aOR = 1.78, 95% CI: 1.56–2.03, *P* < 0.001), though with a relatively modest effect compared with TyG (Table 2). These findings suggest that the TyG index may represent a more sensitive predictor of GDM risk than TG/HDL ratio alone.

**Table 2:** Logistic regression of risk factors for GDM

Variables	aOR (95% CI)	*P*-value
FIN	1.01(1.00,1.01)	< 0.001***
1hPIN	1.01(1.00,1.01)	< 0.001***
2hPIN	1.00(1.00,1.00)	< 0.001***
HOMA-IR	1.53(1.43,1.64)	< 0.001***
HOMA-IS	1.00(1.00,1.00)	0.059
Homa-β	1.00(1.00,1.00)	0.237
QUICKI	0.02(0.01,0.03)	< 0.001***
TyG	4.07(3.34,4.95)	< 0.001***
TyG-BMI	1.01(1.01,1.01)	< 0.001***
TG/HDL ratio	1.78(1.56,2.03)	< 0.001***

Adjusted: age, BMI, gravidity, and parity were adjusted

*P < 0.05; **P < 0.01; ***P < 0.001

**Fig. 1:**
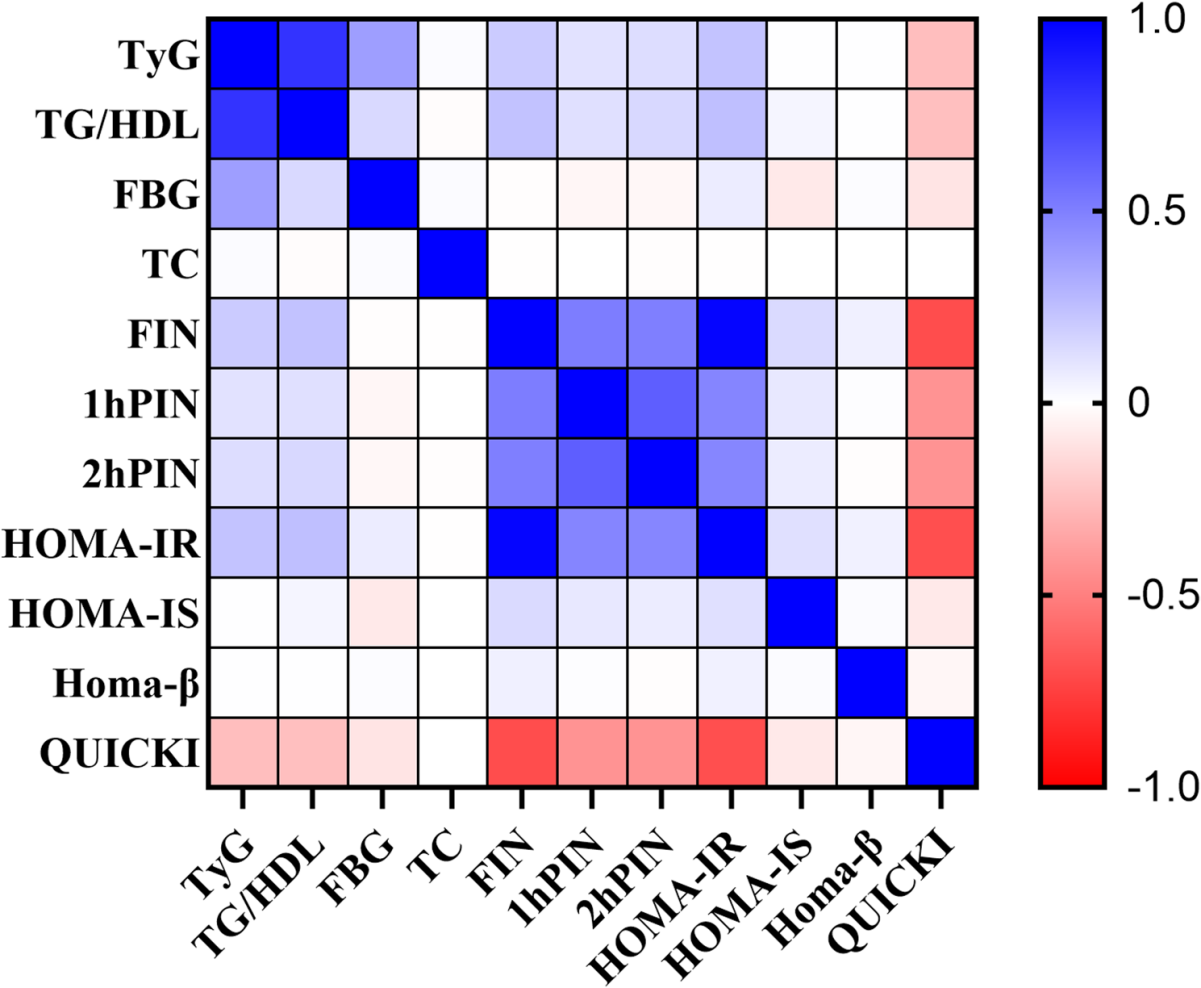
Correlation matrix of the TyG index, TG/HDL ratio and metabolic indicators in early pregnancy. The x- and y-axes list the TyG index, TG/HDL ratio, BMI (kg/m²), FPG, TG, TC, HDL and LDL (all in mmol/L), as well as insulin-resistance indices. Cell colors indicate Pearson correlation coefficients (r), as shown by the accompanying color bar. All correlations are calculated in the full cohort (*n* = 4,239)

In Fig. 1, both axes list the metabolic variables, with FPG, TG, TC, HDL and LDL expressed in mmol/L and BMI expressed in kg/m², while the TyG index and TG/HDL ratio are dimensionless indices. The color scale represents the Pearson correlation coefficient (r), and all correlations are based on the full analytic cohort (*n* = 4,239).

**Fig. 2:**
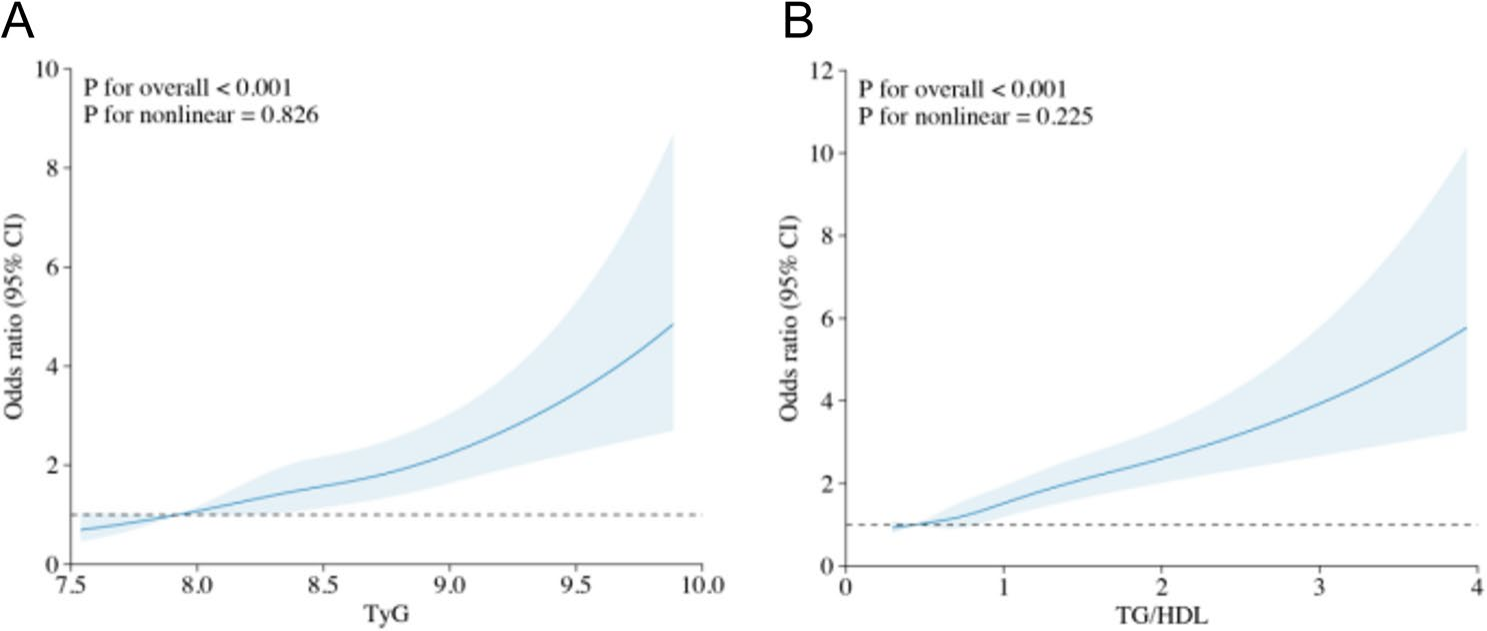
Dose-response relationship of the TyG index and TG/HDL ratio with GDM risk based on restricted cubic spline (RCS) logistic regression (*n* = 4,239). **A** TyG index (x-axis, dimensionless) versus adjusted odds ratio (OR) for GDM (y-axis, log scale). **B** TG/HDL ratio (x-axis, dimensionless) versus adjusted OR for GDM (y-axis, log scale). Three knots were placed at the 10th, 50th and 90th percentiles of each index, as indicated by the tick marks on the x-axis. Solid lines represent multivariable-adjusted ORs (adjusted for maternal age, pre-pregnancy BMI, gravidity and parity), and shaded areas show 95% confidence intervals. The median value of each index was used as the reference (OR = 1.0)

Restricted cubic spline regression further revealed a near-linear positive association between both TyG and TG/HDL ratio and the risk of GDM (Fig. 2). For TyG, the risk started to increase substantially when the index exceeded 8.5, while TG/HDL ratio demonstrated a gradual risk elevation across the entire distribution. No significant non-linear associations were observed (P for nonlinearity > 0.05).

A total of 4,239 pregnant women were categorized into four groups according to the TyG index and TG/HDL ratio (Table 3). Significant differences in demographic and biochemical characteristics were observed across the groups.

**Table 3:** Baseline characteristics and metabolic parameters by TyG index and TG/HDL ratio groups

Variables		Group1 (*n* = 1592) M(P25,P75)/*N*(%)	Group2 (*n* = 322) M(P25,P75)/*N*(%)	Group3 (*n* = 478) M(P25,P75)/N(%)	Group4 (*n* = 1847) M(P25,P75)/N(%)	*P*-value
Demographic characteristics						
Maternal age(years)		30.00(27.00,33.00)	31.00(28.00,33.00)	30.00(27.00,33.00)	31.00(28.00,34.00)	< 0.001***
pre-BMI (kg/m2)		23.12(20.45,26.08)	22.74(20.15,26.11)	24.22(21.72,27.33)	24.22(21.56,27.41)	< 0.001***
Gravidity	1	657(41.3)	122(37.9)	181(37.9)	551(29.8)	< 0.001***
	2	453(28.5)	91(28.3)	150(31.4)	608(32.9)	
	≥ 3	482(30.2)	109(33.8)	147(30.7)	688(37.3)	
Parity	0	533(33.5)	110(34.2)	150(31.4)	567(30.7)	0.003**
	1	753(47.3)	147(45.7)	212(44.4)	806(43.6)	
	≥ 2	306(19.2)	65(20.1)	116(24.2)	474(25.7)	
GDM		234(14.7)	88(27.3)	69(14.4)	528(28.6)	< 0.001***
Biochemical indicators						
FPG(mmol/L)		4.46(4.20,4.73)	4.80(4.56,5.12)	4.32(4.04,4.61)	4.66(4.38,4.99)	< 0.001***
TG(mmol/L)		1.02(0.89,1.16)	1.43(1.33,1.56)	1.25(1.15,1.35)	1.79(1.56,2.12)	0.000***
TC(mmol/L)		4.60(4.17,5.10)	5.34(4.90,5.95)	4.29(3.91,4.75)	4.87(4.37,5.45)	< 0.001***
HDL(mmol/L)		1.75(1.56,1.97)	2.06(1.90,2.23)	1.35(1.20,1.49)	1.51(1.31,1.71)	< 0.001***
LDL(mmol/L)		2.17(1.84,2.56)	2.45(2.08,3.00)	2.27(1.91,2.66)	2.49(2.07,2.94)	< 0.001***
FIN(µU/ml)		40.10(29.85,59.18)	42.35(32.06,57.00)	52.52(36.65,75.11)	57.90(41.39,80.85)	< 0.001***
1hPIN(µU/ml)		340.00(226.48,507.65)	350.55(255.78,515.13)	392.80(238.40,613.05)	408.80(266.45,601.30)	< 0.001***
2hPIN(µU/ml)		284.30(185.04,426.40)	294.20(183.58,459.25)	330.85(195.98,498.54)	361.20(230.80,542.42)	< 0.001***
HOMA-IR		1.16(0.84,1.69)	1.27(0.95,1.71)	1.47(1.02,2.14)	1.72(1.19,2.44)	< 0.001***
HOMA-IS		132.58(87.56,204.52)	109.76(73.69,163.84)	171.04(114.77,267.81)	154.28(102.28,234.19)	0.007**
Homa-β		5.98(3.47,9.96)	5.38(3.49,9.88)	7.49(4.40,11.65)	7.24(4.51,11.91)	< 0.001***
QUICKI		0.70(0.63,0.78)	0.69(0.63,0.75)	0.66(0.59,0.73)	0.63(0.57,0.70)	< 0.001***
TyG-BMI		189.40(166.43,213.28)	195.94(174.52,223.98)	202.06(182.19,228.49)	214.08(190.07,243.74)	< 0.001***

Group 1: TyG < 8.5 & TG/HDL ratio < 0.8; Group 2: TyG ≥ 8.5 & TG/HDL ratio < 0.8; Group 3: TyG < 8.5& TG/HDL ratio ≥ 0.8; Group 4: TyG ≥ 8.5 & TG/HDL ratio ≥ 0.8. All lipid parameters (TG, TC, HDL, LDL) and FPG are expressed in mmol/L. TG/HDL ratio is unitless

*P < 0.05; **P < 0.01; ***P < 0.001

Maternal age was slightly higher in Groups 2–4 compared with Group 1 (*P* < 0.001). Pre-pregnancy BMI showed a progressive increase from Group 2 (median 22.74 kg/m²) to Groups 3–4 (median 24.22 kg/m²; *P* < 0.001). Gravidity ≥ 3 was more prevalent in Group 4 (37.2%) than in Group 1 (20.2%), while parity ≥ 2 was also more frequent in Group 4 (25.7% vs. 19.2%; *P* = 0.003).

The incidence of GDM varied markedly across groups, ranging from 14.7% in Group 1 to 28.6% in Group 4 (*P* < 0.001).

With respect to biochemical indicators, women in Group 4 exhibited the most adverse metabolic profile, with significantly elevated FPG (4.66 mmol/L), TG (1.79 mmol/L), LDL (2.49 mmol/L), and fasting insulin (57.9 µU/mL), together with higher 1 h- and 2 h-postload insulin levels (*P* < 0.001 for all). In contrast, HDL progressively decreased from Group 1 (1.75 mmol/L) to Group 4 (1.51 mmol/L; *P* < 0.001). Markers of insulin resistance also showed a worsening trend across groups, as HOMA-IR increased from 1.16 in Group 1 to 1.62 in Group 4 (*P* < 0.001), while insulin sensitivity indices declined, with HOMA-IS dropping from 132.6 to 134.8 (*P* < 0.001) and QUICKI from 0.70 to 0.65 (*P* < 0.001).

Table 4 presents the associations between the four groups and the risk of GDM. In the crude model, women in Group 2 (OR = 2.18, 95% CI: 1.65–2.89, *P* < 0.001) and Group 4 (OR = 2.32, 95% CI: 1.96–2.76, *P* < 0.001) had significantly higher odds of developing GDM compared with Group 1, whereas no significant difference was observed for Group 3 (OR = 0.98, 95% CI: 0.73–1.31, *P* = 0.89).

**Table 4:** Association of combined TyG index and TG/HDL ratio with GDM risk

	Crude Model		Adjusted Model	
	OR (95%CI)	*P* value	OR (95%CI)	*P* value
Group1	Ref		Ref	
Group2	2.18(1.65,2.89)	< 0.001***	2.7(1.55,2.75)	< 0.001***
Group3	0.98(0.73,1.31)	0.89	0.95(0.71,1.27)	0.71
Group4	2.23(1.96,2.76)	< 0.001***	2.11(1.77,2.52)	< 0.001***

Crude model: none was adjusted; Adjusted model: age, BMI, gravidity, and parity were adjusted. Group 1 (low TyG / low TG–HDL): TyG < 8.5 AND TG/HDL ratio < 0.8

Group 2 (high TyG / low TG–HDL): TyG ≥ 8.5 AND TG/HDL ratio < 0.8

Group 3 (low TyG / high TG–HDL): TyG < 8.5 AND TG/HDL ratio ≥ 0.8

Group 4 (high TyG / high TG–HDL): TyG ≥ 8.5 AND TG/HDL ratio ≥ 0.8

*P < 0.05; **P < 0.01; ***P < 0.001

After adjustment for maternal age, pre-pregnancy BMI, gravidity, and parity, the associations remained robust. Group 2 (OR = 2.07, 95% CI: 1.55–2.75, *P* < 0.001) and Group 4 (OR = 2.11, 95% CI: 1.77–2.52, *P* < 0.001) continued to show significantly elevated risks, while Group 3 did not differ from Group 1 (OR = 0.95, 95% CI: 0.71–1.27, *P* = 0.71).

Overall, participants with both high TyG index and high TG/HDL ratio (Group 4) were characterized by higher pre-pregnancy BMI, more adverse lipid and glucose parameters, and the highest prevalence of GDM, indicating that these two indices show a combined association with metabolic risk during early pregnancy. Notably, women in Group 2 (high TyG with low TG/HDL ratio) also showed an increased risk, suggesting that the TyG index may be more strongly associated with GDM risk than TG/HDL ratio alone.

In the stratified analysis, a higher TyG index (≥ 8.5) was consistently associated with an increased risk of GDM across different TG/HDL ratio categories. Among women with TG/HDL ratio < 0.8, those with TyG ≥ 8.5 had a 2.18-fold higher risk of GDM (OR = 2.18, 95% CI: 1.65–2.89, *P* < 0.001), while the risk increased to 2.37-fold in those with TG/HDL ratio ≥ 0.8 (OR = 2.37, 95% CI: 1.80–3.12, *P* < 0.001). In contrast, elevated TG/HDL ratio alone did not significantly increase GDM risk within the low TyG subgroup. (Table 5)

**Table 5:** Stratified risk analysis of GDM by TyG index and TG/HDL ratio subgroups

Subgroup	GDM	
	OR (95%CI)	*P* value
TG/HDL ratio < 0.8 (*n* = 1914)		
TyG index < 8.5	Ref	
TyG index ≥ 8.5	2.18(1.65,2.89)	< 0.001***
TG/HDL ratio ≥ 0.8 (*n* = 1914)		
TyG index < 8.5	Ref	
TyG index ≥ 8.5	2.37(1.80,3.12)	< 0.001***
TyG index < 8.5 (*n* = 2070)		
TG/HDL ratio < 0.8	Ref	
TG/HDL ratio ≥ 0.8	0.98(0.74,1.31)	0.9
TyG index ≥ 8.5 (*n* = 2169)		
TG/HDL ratio < 0.8	Ref	
TG/HDL ratio ≥ 0.8	1.06(0.82,1.39)	0.64

Boldface indicates statistical significance (*P* < 0.05)

*P < 0.05; **P < 0.01; ***P < 0.001

**Fig. 3:**
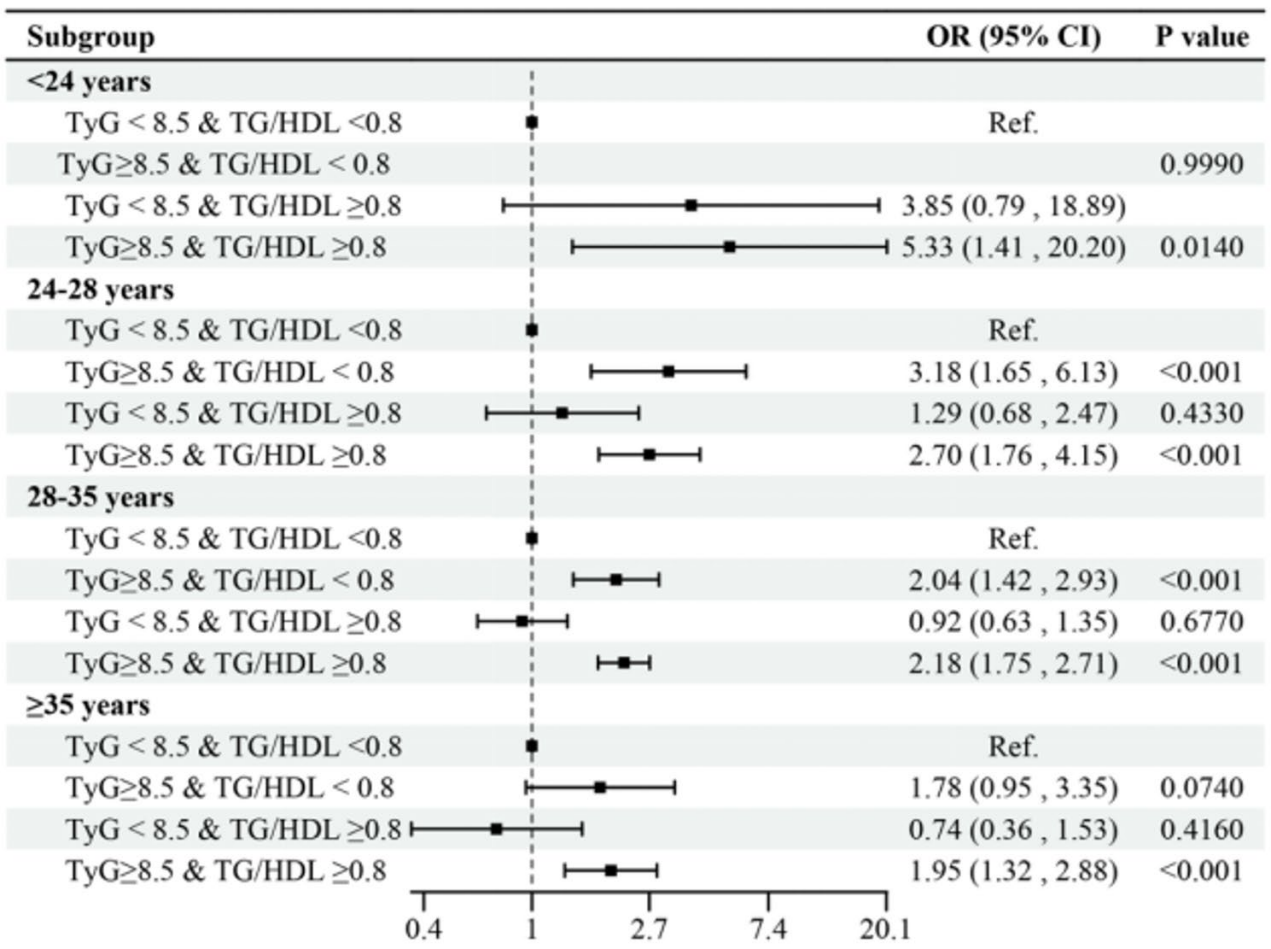
Age-associated risk of TyG index and TG/HDL ratio for GDM. The x-axis shows maternal age categories (years), and the y-axis shows adjusted odds ratios (ORs) for GDM on a logarithmic scale. Number of participants: < 24 years (n = 136); 24–28 years (n = 873); 28–35 years (n = 2,560); ≥ 35 years (n = 670). Dots and lines represent ORs and 95% confidence intervals, respectively

Further stratification by maternal age revealed a consistent joint association of TyG index and TG/HDL ratio. In women < 24 years, the combination of TyG ≥ 8.5 and TG/HDL ratio ≥ 0.8 conferred the highest risk (OR = 5.33, 95% CI: 1.41–20.20, *P* = 0.014). Similarly, significant associations were observed in the 24–28 years (OR = 2.70, 95% CI: 1.76–4.15, *P* < 0.001) and 28–35 years groups (OR = 2.18, 95% CI: 1.75–2.71, *P* < 0.001). In women aged ≥ 35 years, the effect size was attenuated but remained statistically significant (OR = 1.95, 95% CI: 1.32–2.88, *P* < 0.001). (Fig. 3)

### Mediation and correlation analysis

**Fig. 4:**
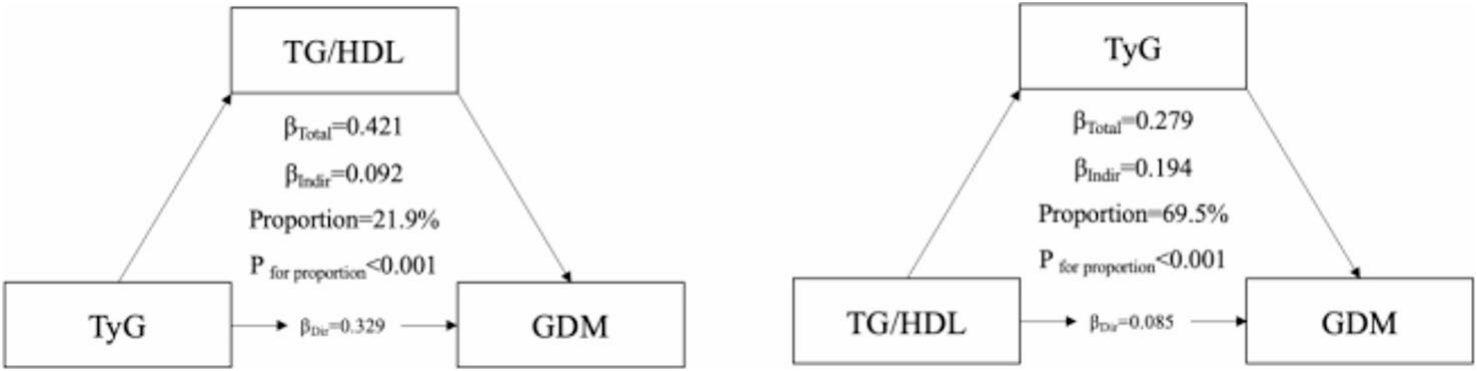
Mutual statistical relationships between the TyG index, TG/HDL ratio and GDM. The diagram summarizes the partitioning of shared variance between the TyG index and TG/HDL ratio in relation to GDM in the full cohort (n = 4,239). Arrows represent regression paths adjusted for maternal age, pre-pregnancy BMI, gravidity and parity. Because both indices were measured cross-sectionally and share triglycerides as a common component, this figure illustrates statistical interdependence rather than temporal or biological mediation

Figure 4 illustrates statistical interdependence between TyG and TG/HDL ratio rather than causal mediation. Because both indices were measured cross-sectionally and share triglycerides as a common metabolic component in their definitions, the “mediation” analysis primarily reflects mathematical coupling and partitioning of shared variance, rather than a temporal or biological pathway. In other words, the reported indirect-effect proportions should not be interpreted as true mechanistic mediation, but as a descriptive way of quantifying how much of the association of each index with GDM can be statistically attributed to overlapping TG-related information. In practical terms, these patterns are consistent with the idea that impaired glucose metabolism (reflected by TyG) and lipid imbalance (captured by TG/HDL ratio) often coexist in early pregnancy and may jointly signal metabolic vulnerability related to GDM. The TG/HDL ratio statistically accounted for 21.9% of the association between TyG and GDM, while TyG accounted for 69.5% of the statistical association between TG/HDL ratio and GDM.

**Fig. 5:**
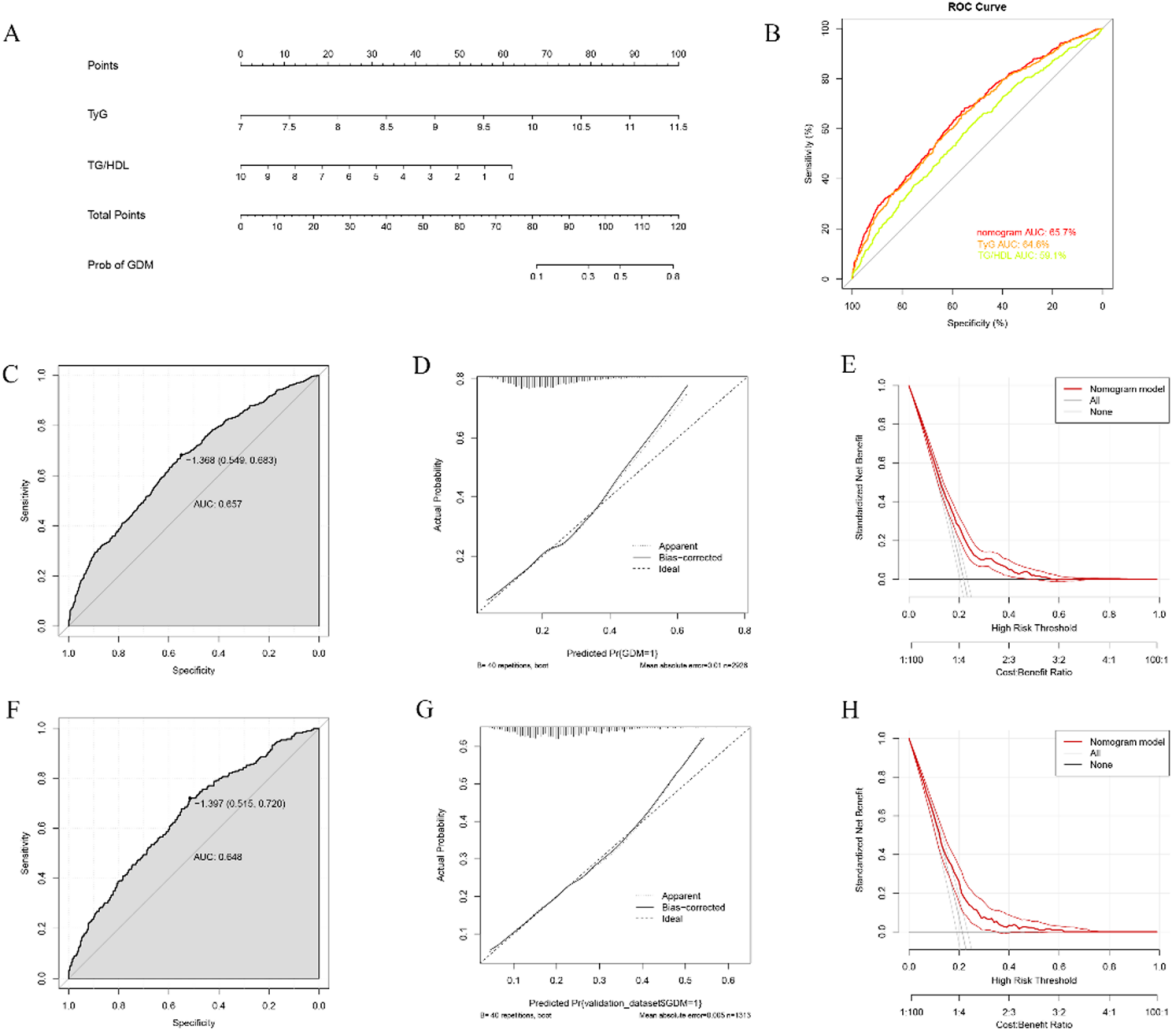
**A** Nomogram incorporating maternal age, pre-pregnancy BMI, gravidity, parity, the TyG index and the TG/HDL ratio for individualized prediction of GDM in the training cohort (70% of the sample, *n* = 2,967). The top axis shows the point score assigned to each predictor, and the bottom axes indicate the total points and corresponding predicted probability of GDM. **B** Receiver operating characteristic (ROC) curves comparing the discriminative performance of the nomogram, TyG index and TG/HDL ratio in the training cohort (x-axis: 1- specificity; y-axis: sensitivity). **C**, **F** ROC curves of the nomogram model in the training (**C**, *n* = 2,967) and validation (F, 30% of the sample, *n* = 1,272) cohorts, with corresponding AUC values. **D**, **G** Calibration plots of the nomogram in the training (**D**) and validation (**G**) cohorts (x-axis: predicted probability of GDM; y-axis: observed probability), showing good agreement between predicted and observed risks. **E**, **H** Decision curve analysis (DCA) in the training (**E**) and validation (**H**) cohorts (x-axis: threshold probability; y-axis: standardized net benefit), demonstrating the net clinical benefit of the nomogram compared with the “treat-all” and “treat-none” strategies

Figure 5A illustrates the construction of a nomogram model incorporating the triglyceride-glucose (TyG) index and the triglyceride-to-HDL cholesterol (TG/HDL) ratio to predict the risk of gestational diabetes mellitus (GDM). Each variable is assigned a point score, and the total points correspond to an estimated probability of GDM, allowing for individualized risk assessment. To explore whether TyG and TG/HDL ratio contribute beyond basic clinical characteristics, we compared three prespecified models. Building on the mediation analysis that demonstrated statistical interdependence between TyG and TG/HDL ratio (Fig. 4), this nomogram was developed to translate these combined metabolic effects into a practical, individualized risk prediction tool. The addition of TyG (Model 1) and TG/HDL ratio (Model 2) showed only modest and non-substantial incremental improvement relative to Model 0, indicating that these indices provide complementary rather than transformative predictive information. As a minimal additional analysis, we also performed univariate ROC analyses to benchmark TyG and TG/HDL against conventional measures (BMI and FPG) (Table S1). In the validation set, the AUCs were 0.562 for BMI and 0.706 for FPG, compared with 0.634 for TyG and 0.579 for TG/HDL.This stepwise enhancement remained limited, and the final nomogram still exhibited modest discrimination overall.

As shown in Fig. 5B, the nomogram model demonstrated superior discriminative ability compared to the individual predictors. The nomogram yielded an AUC of 0.657 in the training set and 0.648 in the internal validation set, higher than that of the TyG index (0.646) and TG/HDL ratio (0.591), suggesting improved classification performance.

In the training cohort (Fig. 5C), the nomogram achieved a sensitivity of 68.3% and specificity of 54.9% at the optimal cutoff value of -1.368. In the validation cohort (Fig. 5F), the model yielded an AUC of 0.648, with a sensitivity of 72.0% and specificity of 51.5% at a threshold of -1.397, indicating stable performance across datasets.

Calibration plots (Fig. 5D and G) showed that the bias-corrected curves closely aligned with the ideal diagonal, indicating good agreement between predicted and observed probabilities. The mean absolute error was 0.010 in the training set and 0.005 in the validation set, supporting the model’s reliable calibration.

Decision curve analysis (Figs. 5E and H) demonstrated that the nomogram provided a greater standardized net benefit than either the “treat-all” or “treat-none” strategies within a clinically relevant range of threshold probabilities (0.05–0.45). This suggests that the model has potential value in supporting clinical decision-making and risk stratification. In simple terms, within this threshold range, using the nomogram to guide early screening would identify more women who will develop GDM while avoiding unnecessary interventions in those at genuinely low risk.

## Discussion

In this large retrospective cohort of over 4,000 pregnant women, we demonstrated that both the triglyceride-glucose (TyG) index and the triglyceride-to-HDL cholesterol (TG/HDL) ratio in early pregnancy were significantly and independently associated with the subsequent development of gestational diabetes mellitus (GDM). Importantly, our findings highlight a combined association when these two indices were jointly elevated, with the highest GDM risk observed among women with both high TyG and high TG/HDL ratio. Moreover, mediation analysis revealed reciprocal relationships between the two indices, and the integration of both into a nomogram yielded only a small numerical increase in discrimination, with overall performance remaining in the fair range, consistent with a modest overall predictive ability.

Previous studies have consistently identified insulin resistance as the core pathophysiological mechanism underlying GDM [28, 29]. However, traditional reference methods such as the hyperinsulinaemic–euglycaemic clamp are invasive and time-consuming, and although HOMA-IR is non-invasive, it still requires fasting insulin measurement and is therefore less feasible for large-scale routine screening in pregnancy [21]. In this context, the TyG index, derived from simple fasting glucose and triglycerides, has emerged as a practical surrogate of insulin resistance and has been validated in predicting diabetes and cardiovascular outcomes [30, 31]. Similarly, the TG/HDL ratio reflects atherogenic dyslipidemia and impaired lipid metabolism, which have also been linked to insulin resistance and cardiometabolic risk [32, 33]. Our study extends these findings to the field of obstetric metabolism, confirming that both indices are independently predictive of GDM when measured in early gestation.

From a pathophysiological perspective, insulin resistance and chronic low-grade inflammation during pregnancy are often accompanied by a dyslipidaemic profile characterized by increased triglycerides and reduced HDL concentrations. Recent evidence highlights the bidirectional crosstalk between inflammation and insulin resistance, in which inflammatory cytokines impair insulin signalling and subsequently promote dyslipidaemia, particularly elevated TG and reduced HDL [34]. This interaction further supports the biological plausibility of using TG- and HDL-related indices as early indicators of metabolic dysfunction in pregnancy. In our cohort, women at higher GDM risk, particularly those with concurrently elevated TyG and TG/HDL ratio(Group 4), exhibited this more adverse lipid pattern in early pregnancy, with higher TG and lower HDL levels and a clearly elevated TG/HDL ratio. This is consistent with the concept that hyperglycaemia and insulin resistance tend to cluster with atherogenic dyslipidaemia, providing biological plausibility for the associations observed in our study. In addition, ultrastructural studies have reported distinctive placental alterations in GDM pregnancies complicated by macrosomia, further supporting a link between maternal metabolic dysregulation and excessive fetal growth [5].

Our study further characterizes the combined association between TyG and TG/HDL ratio, showing that women with both elevated markers had the highest risk. This combined stratification provides exploratory phenotypic information, but does not represent a clinically transformative or clearly superior tool compared with traditional markers, consistent with previous literature.

Notably, women with elevated TyG but low TG/HDL ratio(Group 2) also had an increased risk, suggesting that glucose–triglyceride interactions captured by TyG may be more centrally associated with GDM risk than those captured by TG/HDL ratio alone. This aligns with mechanistic evidence indicating that hepatic and adipose insulin resistance, as reflected by elevated fasting triglycerides and glucose, may precede pancreatic β-cell dysfunction and overt dysglycemia during pregnancy [35, 36]. However, given the modest effect sizes and limited AUC improvement, this four-group stratification should be regarded as exploratory and descriptive, rather than a clinically impactful innovation.

The restricted cubic spline analysis further revealed that GDM risk increased almost linearly with rising TyG and TG/HDL ratio, with a critical inflection point for TyG at approximately 8.5. This threshold may represent a clinically relevant cutoff for risk stratification, complementing conventional screening methods. Moreover, stratified analyses demonstrated that the joint predictive value of TyG and TG/HDL ratio was particularly pronounced in younger women (< 35 years), whereas the relative effect size was attenuated in those ≥ 35 years. This pattern suggests that early metabolic disturbances may be more strongly associated with GDM among younger women, while age-related β-cell insufficiency may represent a relatively more prominent correlate in older pregnancies [37, 38].

Another aspect of our analysis involved exploratory variance-partitioning approaches to examine the statistical interdependence between TyG and TG/HDL ratio. Because both indices were measured at the same time point and share triglycerides as a common metabolic component, these findings do not reflect temporal or biological mediation. Instead, they illustrate overlapping metabolic pathways captured by the two indices. TyG statistically accounted for a larger proportion of shared variance with GDM than TG/HDL ratio, suggesting that TyG may reflect a broader spectrum of insulin-resistance–related dysregulation rather than indicating a causal pathway. These findings indicate that while both indices capture overlapping metabolic pathways, TyG may be the more strongly correlated with in GDM risk prediction. This also supports the notion that TyG and TG/HDL ratio represent complementary but non-redundant markers of insulin resistance and dyslipidemia. It is important to note, however, that formal mediation analysis relies on strong assumptions, including temporal precedence of the mediator and the absence of unmeasured confounding between the exposure, mediator, and outcome. Because TyG and TG/HDL ratio were measured simultaneously and are algebraically related through triglycerides, these assumptions are not met in our study. Accordingly, the mediation results should be interpreted as a purely statistical decomposition of correlated markers rather than evidence of distinct biological pathways linking TyG, TG/HDL ratio, and GDM.

From a clinical perspective, the development of a nomogram incorporating both TyG and TG/HDL ratio offers an exploratory and individualized early-pregnancy risk assessment tool rather than a definitive decision aid. The nomogram demonstrated modest discrimination (AUC 0.657 in the training set and 0.648 in the internal validation set), with the validation AUC serving as the primary estimate of model performance. Calibration was acceptable in both datasets.

Although the nomogram performed slightly better than the individual markers, the absolute improvement was small. The model demonstrated modest discrimination (AUC ~ 0.65) and acceptable calibration, with only slight improvement over the baseline clinical model. These univariate AUC benchmarks provide additional context for our main findings, supporting that TyG and TG/HDL offer complementary rather than transformative discrimination beyond conventional predictors. Importantly, we additionally performed minimal univariate ROC/AUC benchmarking against conventional predictors (BMI and fasting plasma glucose), which indicated that TyG and TG/HDL alone did not outperform these conventional measures; therefore, the model should be viewed as exploratory rather than a standalone decision tool.

Although the AUC values indicate only modest discrimination and do not approach diagnostic performance, the nomogram may still offer exploratory value because it can be applied in early pregnancy—well before the conventional 24–28-week OGTT screening window. This suggests potential value in primary prevention, as the model may help identify women who could be considered for earlier lifestyle counselling or more intensive monitoring [39, 40]. Furthermore, given that many women with prior GDM do not complete recommended postpartum glucose follow-up screening in routine practice [41], early identification of high-risk individuals may also help clinicians prioritize postpartum surveillance and long-term preventive strategies. Given that an AUC of approximately 0.65 reflects only fair discrimination, the nomogram should not be viewed as a diagnostic tool but rather as an exploratory early-risk assessment aid that requires further optimization and external validation. Given the relatively high events-per-variable ratio for the six predictors included in the nomogram, and the close agreement between performance in the training and validation sets, substantial overfitting due to model complexity is unlikely. Nonetheless, because both model development and validation were conducted within the same single-center cohort, some degree of optimism in the performance estimates cannot be completely excluded, and external validation in independent populations remains essential. Overall, the contribution of this study lies primarily in providing population-specific evidence from a large Chinese cohort and offering exploratory insights into the combined use of TyG and TG/HDL ratio. We do not claim that these indices substantially improve prediction beyond traditional markers; instead, our findings should be interpreted as incremental and hypothesis-generating, rather than transformative.

Nevertheless, several limitations warrant consideration. First, the study population was derived from a single center in China, which may limit the generalizability of our findings to other ethnicities or healthcare settings. Second, although we adjusted for key clinical risk factors available in our database (maternal age, pre-pregnancy BMI, gravidity, and parity), we were unable to account for other established determinants of GDM, including family history of diabetes, history of GDM in previous pregnancies, detailed dietary patterns, physical activity, smoking and alcohol use, and socioeconomic indicators such as education, income, and occupation. These variables were not systematically collected or contained substantial missing data in the electronic medical records; therefore, they could not be reliably incorporated into the multivariable models. Residual confounding by these unmeasured factors cannot be excluded and may have led to either overestimation or underestimation of the associations between TyG, TG/HDL ratio, and GDM. Third, although the predictive performance of the nomogram was modest (AUC ~ 0.65) and improved only slightly upon the clinical baseline model, the relatively high number of events per predictor and the use of random training/validation splitting with bootstrap-based internal validation reduce, but do not eliminate, the possibility of overfitting. Some optimism in the model’s apparent performance is therefore likely, and external validation in independent cohorts, as well as integration with additional clinical and biochemical parameters (e.g., adipokines, inflammatory markers, or metabolomic signatures), will be required to optimize and confirm the model before considering any clinical implementation. Fourth, we did not perform formal head-to-head performance comparisons (e.g., AUC or reclassification statistics). Therefore, the precise magnitude of the incremental value of these indices over single conventional markers remains uncertain and should be determined in future studies. Finally, the observational design precludes causal inference, and prospective studies or interventional trials are needed to determine whether modifying lipid–glucose dysregulation in early pregnancy can reduce the incidence of GDM. In addition, we were unable to describe true trimester-wise trajectories or dynamic age-metabolism interactions across gestation within this dataset.

Overall, the contribution of this study lies primarily in providing population-specific evidence from a large Chinese cohort and offering exploratory insights into the combined use of TyG and TG/HDL ratio. We do not claim conceptual novelty, and our findings should be interpreted as incremental rather than transformative.

## Conclusions

In conclusion, this study provides evidence that early-pregnancy TyG index and TG/HDL ratio are strongly and independently associated with subsequent GDM. Their combination provides modest improvement in predictive performance and may help support early exploratory risk stratification rather than serve as a diagnostic tool, particularly among younger pregnant women. These findings provide supportive, exploratory evidence rather than a clinically ready risk-prediction tool. Future external validation and integration with broader clinical markers are required before considering clinical implementation. Future research should focus on validating these findings across diverse populations and exploring the mechanistic pathways linking lipid–glucose metabolism to gestational glucose intolerance.

## Supplementary Information

Supplementary Material 1.

Supplementary Material 2.

## Data Availability

Data are available upon reasonable request. The datasets generated during and/or analysed during the current study are available upon reasonable request through the corresponding author.
