# Supplementary figures and images for: Nomogram incorporating TyG index and TG/HDL ratio for early prediction of gestational diabetes mellitus
Source: BMC Pregnancy Childbirth. 2026 Feb 18;26:575. doi: 10.1186/s12884-026-08732-y (PMC13202934; doi:10.1186/s12884-026-08732-y)

Figure S1. Flowchart of participant selection.


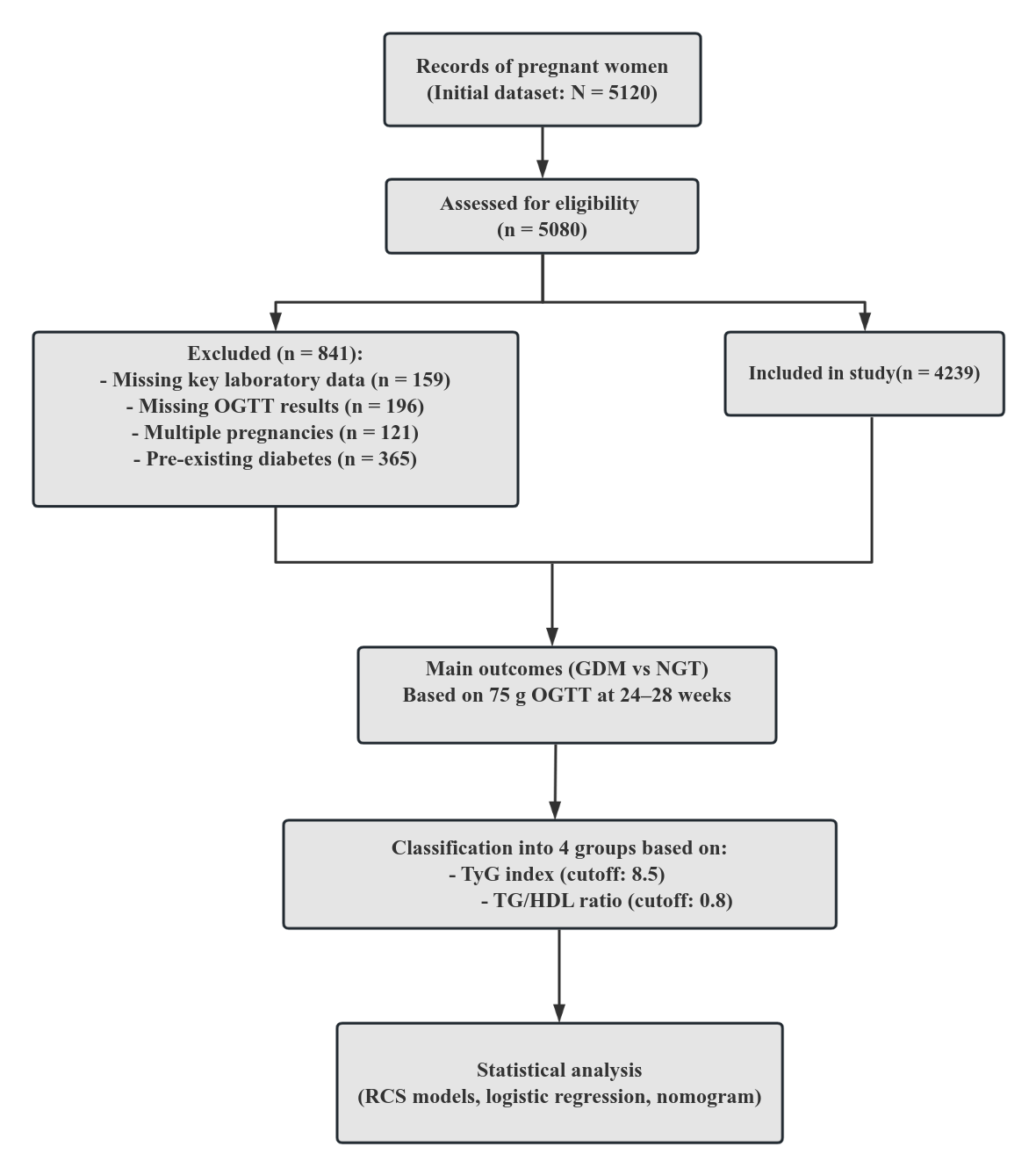

Supplement: Supplementary file 1 — Supplementary Material 1. [file 12884_2026_8732_MOESM1_ESM.docx]
