# Supplementary material for: Nomogram incorporating TyG index and TG/HDL ratio for early prediction of gestational diabetes mellitus
Source: BMC Pregnancy Childbirth. 2026 Feb 18;26:575. doi: 10.1186/s12884-026-08732-y (PMC13202934; doi:10.1186/s12884-026-08732-y)
Supplement: Supplementary file 2 — Supplementary Material 2. [file 12884_2026_8732_MOESM2_ESM.docx]

| Table S1 Univariate AUCs (95% CIs) of BMI, FPG, TyG, and TG/HDL for predicting gestational diabetes mellitus in the training and validation sets (with combined models shown for reference). | | | |
| --- | --- | --- | --- |
| Predictors | Training AUC (95% CI) | Validation AUC (95% CI) | P value (DeLong) vs Model 1 |
| BMI | 0.565 (0.541, 0.589) | 0.562 (0.524, 0.599) | <0.001 |
| FPG | 0.704 (0.680, 0.728) | 0.706 (0.671, 0.742) | 0.035 |
| TyG | 0.648 (0.624, 0.672) | 0.634 (0.596, 0.672) | <0.001 |
| TG/HDL | 0.589 (0.564, 0.614) | 0.579 (0.539, 0.618) | <0.001 |
| Model1 (BMI+FPG) | 0.715 (0.691, 0.739) | 0.716 (0.680, 0.752) | Reference |
| Model2 (TyG+TG/HDL) | 0.661 (0.637, 0.685) | 0.639 (0.602, 0.676) | <0.001 |
| Model3 (All combined) | 0.722 (0.699, 0.745) | 0.722 (0.687, 0.757) | 0.423 |
| AUCs (95% CIs) were obtained from ROC analyses in the training and validation sets. Model 1 includes BMI and FPG; Model 2 includes TyG and TG/HDL; Model 3 includes BMI, FPG, TyG, TG/HDL. P values were calculated using DeLong tests comparing each predictor/model with Model 1 (BMI+FPG) in the validation set (Model 1 as the reference). AUCs (95% CIs) were derived from ROC analyses in the training (n = 2,967) and internal validation (30% of the sample, n = 1,272) cohorts. P values were based on DeLong tests versus Model 1 (BMI+FPG) in the validation cohort. | | | |
